# Prediction of Ammonia Mitigation Efficiency in Sodium Bisulfate-Treated Broiler Litter Using Artificial Neural Networks

**DOI:** 10.3390/ani16020210

**Published:** 2026-01-10

**Authors:** Busra Yayli, Ilker Kilic

**Affiliations:** Department of Biosystems Engineering, Faculty of Agriculture, Bursa Uludag University, 16059 Bursa, Türkiye; ikilic@uludag.edu.tr

**Keywords:** ammonia mitigation, artificial neural networks, broiler litter, environmental management, machine learning

## Abstract

The increasing demand for poultry meat, driven by its favorable nutritional profile, including low cholesterol and high protein content, has resulted in intensified production volumes and, consequently, elevated ammonia (NH_3_) emissions. Artificial intelligence-based predictive approaches offer an effective alternative to conventional treatment-oriented methods by enabling faster and more accurate estimation of NH_3_ removal performance. This study aimed to predict the ammonia removal efficiency of broiler litter generated during a production cycle under controlled laboratory-scale conditions using artificial neural networks (ANNs) trained with different learning algorithms. Four ANN models were developed based on the Levenberg–Marquardt (LM), Fletcher–Reeves (FR), Scaled Conjugate Gradient (SCG), and Bayesian Regularization (BR) algorithms. The results showed that the LM-based model with 12 hidden neurons achieved the highest predictive performance (R^2^ = 0.9777; MSE = 0.0033; RMSE = 0.0574; MAPE = 0.0833), while the BR-based model with 10 neurons showed comparable accuracy. In comparison with the FR and SCG models, the LM algorithm demonstrated superior predictive accuracy and generalization capability. Overall, the findings suggest that ANN-based modeling is a reliable, data-informed approach for estimating NH_3_ removal efficiency, providing a potential decision-support framework for ammonia mitigation strategies in poultry production systems.

## Simple Summary

Intensive poultry production has intensified concerns regarding gaseous emissions, particularly ammonia, which can adversely affect animal welfare and occupational health if not adequately controlled. In recent years, artificial intelligence-based approaches have gained increasing attention as effective tools for predicting gaseous emissions and assessing mitigation performance. This study evaluates the predictive capability of artificial neural network models in estimating ammonia mitigation efficiency in broiler litter treated with sodium bisulfate under controlled laboratory-scale conditions. The findings indicate that AI-based modeling supports data-informed decision-making and contributes to the development of more effective environmental management strategies in intensive poultry production systems.

## 1. Introduction

Poultry meat is increasingly preferred as a nutritious alternative to red meat due to its high protein content and relatively low cholesterol levels [1,2]. However, escalating production intensity has led to substantial waste accumulation and the emission of various pollutant gases arising from continuous animal activity, feed consumption, and respiratory processes. Among these emissions, ammonia (NH_3_) is one of the most prevalent and noxious gases encountered in poultry housing systems, characterized by its pungent odor and corrosive properties. Elevated NH_3_ concentrations not only pose significant environmental challenges but also constitute a critical risk factor adversely affecting animal performance, welfare, and occupational health. Prolonged exposure to NH_3_ can cause severe respiratory irritation and other detrimental health effects, thereby emphasizing the necessity for effective ammonia mitigation strategies.

Since poultry are highly sensitive to fluctuations in indoor environmental conditions, effective control of ammonia emissions and indoor NH_3_ concentrations is essential for maintaining optimal housing environments [3]. Air quality within poultry houses is governed by a complex interplay of factors, including stocking density, animal age, housing design and maintenance, temperature, humidity, ventilation rate, lighting conditions, cage systems, bedding material, and bedding temperature [4].

In litter-based poultry production systems, ammonia generation is primarily associated with the microbial degradation of litter and manure, coupled with the simultaneous excretion of urine and feces. Under these conditions, urea excreted in urine is rapidly hydrolyzed to NH_3_ by urease enzymes present in manure. Consequently, bedding materials should be readily available, non-toxic, porous, cost-effective, and capable of efficiently retaining moisture. Elevated litter moisture levels stimulate microbial activity, thereby enhancing ammonia volatilization. Maintaining dry litter conditions through adequate ventilation and the use of sufficiently absorbent materials is therefore considered a key management practice. In addition, elevated ambient temperatures promote microbial and urease activity, accelerating the decomposition of nitrogenous compounds and increasing NH_3_ release [5,6]. Furthermore, increases in litter pH shift the chemical equilibrium toward the non-ionized NH_3_ form, facilitating its transfer to the gas phase and resulting in higher overall ammonia emissions [7].

Given the multifactorial nature of ammonia generation in poultry houses, a wide range of mitigation strategies has been developed to control air pollutant emissions. Two principal approaches are commonly employed: minimizing emissions at their point of origin and reducing pollutant concentrations within the surrounding environment [8,9]. In poultry production systems, strategies aimed at mitigating NH_3_ emissions can be broadly classified into three categories: (i) reducing nitrogen excretion through dietary modifications, (ii) suppressing NH_3_ generation via the application of chemical additives to the litter, and (iii) removing or recovering nitrogen from contaminated air using technologies such as gas scrubbers or biofiltration systems [10,11].

Among these approaches, litter management practices play a critical role in controlling ammonia emissions by retaining NH_3_ within the litter and limiting its release into the atmosphere. In particular, the application of litter amendments has been widely reported as an effective strategy for ammonia mitigation These amendments primarily act by altering litter pH, inhibiting microbial activity, and suppressing enzymatic processes involved in nitrogen transformation, thereby reducing NH_3_ volatilization and enhancing nitrogen retention within the litter [12,13]. Despite the availability of such mitigation strategies, increasing production intensity necessitates more advanced approaches for managing environmental pollutants. In this context, artificial intelligence-based methods offer innovative and data-driven solutions for the prediction and management of pollutant gas emissions.

Over the past decades, artificial intelligence (AI) has undergone rapid advancement, with artificial neural networks (ANNs) emerging as a widely adopted machine learning approach due to their capacity to process large datasets and capture complex nonlinear relationships [14,15]. Compared with conventional mathematical modeling techniques, ANNs provide efficient and cost-effective solutions across a wide range of applied science and engineering disciplines by substantially reducing computational time. Their application in agriculture and livestock production has expanded considerably, driven by the increasing demand for enhanced operational efficiency, sustainability, and practical applicability. Within the framework of precision livestock production, ANNs are increasingly recognized as essential decision-support tools that improve production performance, support economic and environmental sustainability, and facilitate responsible management practices [16,17,18,19].

**Figure 1:**
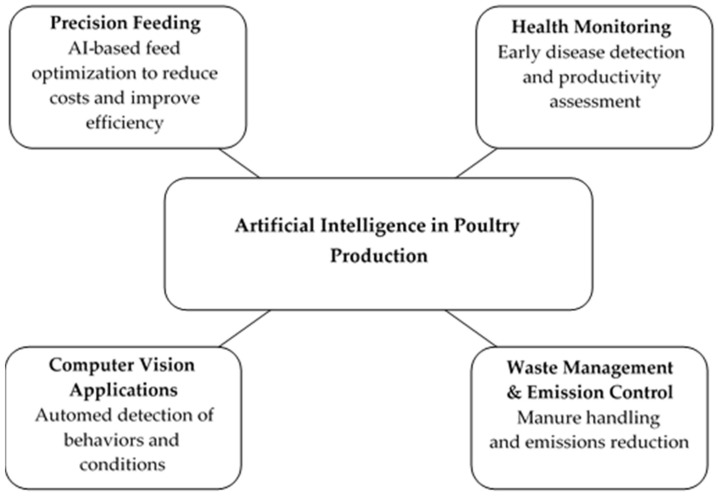
Overview of artificial intelligence applications in poultry production.

Machine learning has been applied across a wide range of livestock production systems, including the optimization of feeding strategies, forecasting performance indicators, monitoring animal health and welfare, waste management, and regulation of environmental conditions [20,21,22,23]. Such applications are essential not only for enhancing resource-use efficiency and reducing environmental impacts but also for meeting the increasing global demand for animal-derived protein. In poultry production, AI-based technologies are increasingly integrated into key operational processes such as feed management, health surveillance, environmental control, waste handling, and performance prediction (Figure 1). Recent studies have demonstrated that AI-driven models can accurately estimate growth performance, improve feed conversion efficiency, and enable early detection of potential health disorders, thereby supporting both economic profitability and environmental sustainability.

Although artificial intelligence applications have become increasingly prevalent in livestock management, the number of studies specifically employing machine learning techniques to predict or mitigate ammonia emissions in poultry production remains limited [24,25,26,27]. Accurate modeling of NH_3_ emissions originating from litter and manure is essential for the development of effective mitigation strategies and for improving air quality within poultry housing systems. While ANN-based approaches have been investigated, the majority of existing research has focused on ammonium nitrogen removal in wastewater treatment systems rather than directly addressing gaseous ammonia emissions in poultry housing environments [28,29,30].

Nevertheless, recent research has demonstrated that artificial intelligence and artificial neural network techniques offer valuable tools for poultry production systems. For example, Yakubu et al. [31] successfully predicted heat stress in Sasso laying hens using pulse and respiration rates as key input variables, achieving high predictive accuracy (R^2^ = 0.966; RMSE = 0.04806). Similarly, Yelmen et al. [32] modeled energy efficiency in broiler farms based on inputs such as feed, fuel, water, labor, and equipment use, reporting strong predictive performance (R^2^ = 0.936). Küçüktopçu and Cemek [33] demonstrated that neurocomputing approaches, including multilayer perceptron and adaptive neuro-fuzzy inference system (ANFIS) models, could estimate ammonia concentrations in poultry houses with high computational efficiency and accuracy. More recently, González-Mora et al. [34] applied machine learning techniques to manure storage systems and identified ambient temperature as a dominant factor influencing ammonia volatilization, reporting an R^2^ value of 0.85.

Despite these advances, intensive poultry production systems continue to face persistent environmental challenges, particularly those associated with manure management and ammonia emissions. Elevated NH_3_ concentrations degrade indoor air quality, adversely affect bird welfare and growth performance, and pose significant occupational health risks. A wide range of litter amendments—including alum, organic and inorganic acids, biochar, and various mineral-based additives—has been investigated for mitigating NH_3_ and other harmful gaseous emissions; however, their reported effectiveness varies considerably depending on application conditions and management practices [35,36,37,38].

Although previous studies have demonstrated that certain additives can effectively reduce ammonia emissions in poultry production under both laboratory and field-scale conditions, a critical gap remains in determining whether the mitigation efficiency observed in laboratory-scale treatments can be reliably predicted using artificial neural networks and other machine learning algorithms.

This study evaluates the predictive capability of artificial neural network models in estimating NH_3_ mitigation efficiency in broiler litter treated with sodium bisulfate under controlled laboratory-scale conditions. By integrating AI-based modeling with controlled laboratory experiments and conducting a comparative evaluation of different ANN training algorithms and neuron configurations, the proposed approach provides a practical framework for accurate prediction of treatment performance and supports data-informed decision-making for ammonia mitigation management in poultry production systems.

## 2. Materials and Methods

### 2.1. Dataset Preparation

The primary data for this study were obtained from trials conducted at the Agricultural Air Quality Laboratory of Bursa Uludag University. The experimental material consisted of broiler litter collected from three different rearing flocks at a commercially operated broiler farm in Bursa Province, Türkiye, between May and December 2023. The litter originated from broilers raised under uniform management conditions and was collected at or near the end of a single production cycle. All experiments were performed under controlled laboratory-scale conditions using spent broiler litter, without the involvement of live animals or the addition of fresh manure. Rice husk served as the bedding material, and the litter consisted of a homogeneous mixture of bedding material, manure, and feathers.

**Figure 2:**
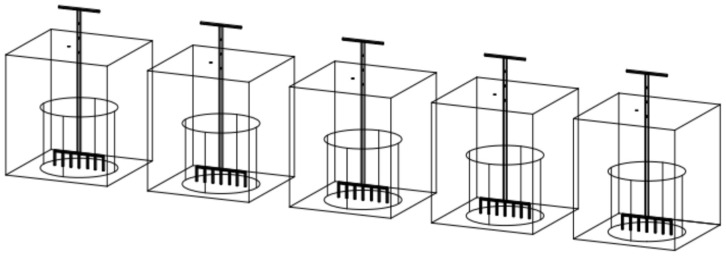
Laboratory-scale experimental setup.

The experimental setup comprised four treatment groups and one control group, implemented using five flux chambers (25 × 25 × 25 cm) (Figure 2). The treatment groups were established by applying sodium bisulfate to litter samples four different application rates: 2.5%, 5.0%, 7.5%, and 10.0% (*w*/*w*). These application rates were selected to represent both low and high dosage ranges based on a comparative assessment of previously reported values in the literature [39,40,41]. Baseline physicochemical properties of the broiler litter, measured prior to sodium bisulfate application across three independent flocks, were as follows: moisture content (14.9 ± 1.24%), pH (8.2 ± 1.85), EC (2755 ± 135.3 µS cm^−1^), TDS (1370.4 ± 67.43 mg L^−1^), and temperature (26.6 ± 0.36 °C).

During the experimental period, the litter was manually mixed once daily using a three-stage rake to ensure homogeneity across litter layers. Air was supplied to each chamber via an inlet pipe connected to the experimental system, and ammonia concentrations were continuously monitored. NH_3_ measurements were conducted under laboratory conditions, with samples collected at 5 min intervals for 1 h over a 14-day experimental period, in the morning, at noon, and in the evening. Ammonia concentrations were analyzed using a multi-gas analyzer (MultiRAE IR Lite, Honeywell, San Jose, CA, USA), which has a measurement range of 0–100 ppm and an accuracy of ±1 ppm [8,42,43]. Before data acquisition, the multi-gas analyzers were calibrated according to the manufacturer’s specifications.

Moisture content of the litter samples was determined using the gravimetric method (Binder ED 23) by oven-drying the samples at 105 °C for 24 h. Electrical conductivity, TDS, and temperature were measured using a portable EC/TDS/temperature meter (Hanna Instruments, Smithfield, RI, USA), while pH was determined using a portable pH meter with a sensitivity of ±0.005 (WTW GmbH, Weilheim, Germany) As reported in our previous study [38], a portion of the primary experimental data has been previously presented. However, the present study focuses on reanalyzing these data to estimate NH_3_ removal efficiency in broiler litter by explicitly considering the combined effects of litter physicochemical properties and sodium bisulfate application—an aspect not addressed in the earlier work. Specifically, this study aims to conduct a comprehensive assessment of changes in litter physicochemical characteristics following the application of varying sodium bisulfate dosages under laboratory-scale conditions. Based on the experimental outcomes, the study further evaluates the potential of artificial neural network models to predict NH_3_ removal efficiency in future treatment scenarios. Overall, this work extends previous research by integrating laboratory-scale litter management experiments with AI-based predictive modeling and by providing a comparative evaluation of ANN training algorithms for ammonia mitigation prediction.

### 2.2. ML-Based Prediction Model

Artificial neural networks are computational modeling frameworks inspired by the functional architecture of the human brain and represent a subset of machine learning techniques within the broader field of artificial intelligence. ANNs consist of interconnected processing units that are capable of learning from data and generalizing acquired knowledge. Their capacity to predict previously unseen values based on historical numerical data makes ANNs particularly effective for modeling complex and nonlinear relationships. Consequently, ANNs have been widely applied across diverse scientific disciplines for tasks such as data classification, pattern recognition, forecasting, performance optimization, and decision-support applications [19,44,45].

**Figure 3:**
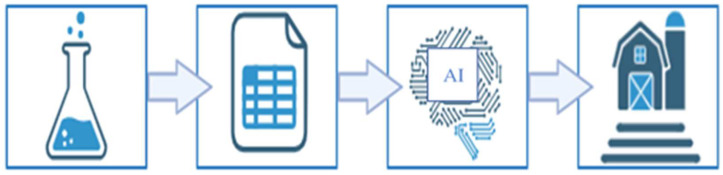
Workflow for AI-driven prediction of field-scale treatment performance from laboratory data.

Implementing gas mitigation strategies in poultry houses through laboratory-scale experiments provides a controlled environment that enables efficient testing while reducing time and labor requirements. However, extrapolating in vitro findings to field conditions may introduce variability and uncertainty due to the complexity of real production systems. Integrating experimental results with artificial intelligence models, particularly artificial neural networks, facilitates the prediction of mitigation performance under practical conditions (Figure 3). The predictive capability of ANNs can accelerate decision-making processes, reduce potential risks, and lower implementation costs, thereby offering substantial economic benefits. In this context, AI-driven modeling serves as a critical link between laboratory-scale research and field-scale applications, supporting the development of sustainable, efficient, reliable, and cost-effective ammonia mitigation strategies.

**Figure 4:**
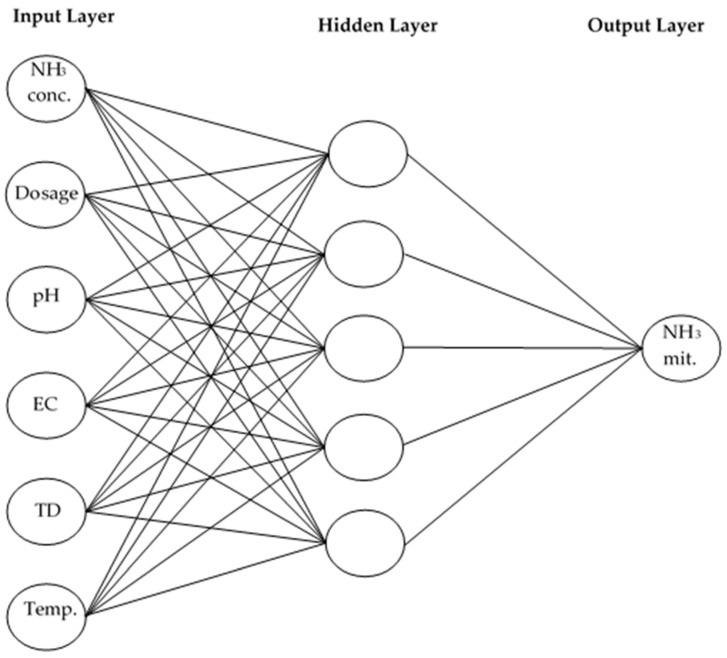
The architecture of the ANN used for predicting NH_3_ mitigation efficiency.

The primary objective of artificial neural networks is to generate accurate output predictions rather than to explicitly describe the underlying relationships between input and output variables. In this study, the output variable was defined as the predicted NH_3_ treatment performance of broiler litter following sodium bisulfate application. The input variables included initial NH_3_ concentration in the litter, sodium bisulfate dosage, pH, electrical conductivity, total dissolved solids, and temperature. Accordingly, ANN models were constructed with six input neurons and one output neuron (Figure 4), consisting of an input layer, a single hidden layer selected for model simplicity and comparability, and an output layer.

Artificial neural networks can be trained using three principal learning paradigms: supervised learning (SL), unsupervised learning (UL), and reinforcement learning (RL). In the present study, a supervised learning (SL) framework was employed. Under this paradigm, output values are generated from input variables, and network weights are iteratively adjusted by minimizing the error between predicted and observed outputs. Because the ANN model was trained using a fully labeled dataset encompassing all input–output pairs, it falls within the supervised learning category. Through this training process, the model learned the underlying input–output relationships present in the experimental data and acquired the capability to generate reliable predictions under defined conditions.

### 2.3. Comparative ANN Modeling

In this study, a feedforward artificial neural network was employed to predict NH_3_ treatment efficiency. In feedforward networks, information propagates unidirectionally from the input layer through one or more hidden layers to the output layer, where the final prediction is generated. To optimize model performance, four feedforward training algorithms were evaluated. The Levenberg–Marquardt (LM) algorithm was selected for its rapid convergence in small- to medium-sized networks; the Fletcher–Reeves (FR) conjugate gradient algorithm is well suited for large-scale optimization problems; the Scaled Conjugate Gradient (SCG) algorithm offers stable convergence with reduced computational cost; and the Bayesian Regularization (BR) algorithm enhances generalization performance by mitigating overfitting.

Each ANN configuration was trained ten times using different random data partitions to assess model robustness and performance variability. Model performance was evaluated using standard statistical error metrics, and the configuration achieving the highest predictive accuracy was selected. In addition, early stopping based on validation error was implemented to further reduce the risk of overfitting.

The dataset was randomly divided into three independent subsets: 60% for training, 15% for validation, and 25% for testing. The model was trained using the training set, while the validation set was employed to monitor performance, prevent overfitting, and select optimal hyperparameters. The test set remained independent throughout model development and was used only after training to provide an objective assessment of the model’s predictive performance.

Linear, sigmoid, rational sigmoid, hyperbolic tangent, and Gaussian functions can be used as activation functions in neural network layers. Among these, the sigmoid and hyperbolic tangent functions are the most commonly applied. The output value of a neuron is determined by its activation function. In this study, the sigmoid function was selected as the activation function because it produces outputs in the range of [0,1] (Equation (1)).

(1) $$\;f\left(x\right)=\frac{1}{1+e^{-x}}$$

In ANNs, input parameters with different units or large variations in numerical values can reduce model performance. To address this issue, all data were normalized to a range between 0 and 1 before training. The normalization process was carried out using Equation (2) [46].

(2) $$\;X_{n=}\;\frac{X_{i}-X_{min}}{\;X_{max}-X_{min}}$$

where X_n_ is the normalized value, X_i_ is the measured value to be normalized, X_min_ is the minimum value in the data set, and X_max_ is the maximum value, respectively.

The performance of artificial neural networks is commonly evaluated using error metrics such as MSE (Mean Squared Error), RMSE (Root Mean Squared Error), and MAPE (Mean Absolute Percentage Error). The equations for calculating these metrics are provided in Equations (3)–(5).

(3) $$MSE=\frac{1}{n}\sum_{i=1}^{n}(y_{i}-{\hat{y}}_{i})$$

(4) $$RMSE=\sqrt{\frac{1}{n}\sum_{i=1}^{n}\left(y_{i}-{\hat{y}}_{i}\right)}$$

(5) $$MAPE=\frac{100}{n}\sum_{i=1}^{n}\left|\frac{y_{i}-{\hat{y}}_{i}}{y_{i}}\right|$$

In the equations, y_i_ denotes the observed (actual) values, ${\hat{y}}_{i}$ denotes the predicted values from the model, *n* is the total number of observations, and i represents the index of each observation (1, 2, …, *n*). All modeling and simulations were conducted in MATLAB R2022a (MathWorks, Natick, MA, USA), ensuring reproducibility and flexibility for different experimental scenarios.

### 2.4. Statistical Analysis

In this study, descriptive statistics (mean, standard deviation, minimum, and maximum values) were used to characterize the experimental dataset obtained from three broiler flocks, and these statistics were calculated using SPSS 29.0.2.0 software. These statistics were used solely to summarize the range and variability of the physicochemical parameters employed as inputs and outputs of the artificial neural network model. Model development and performance evaluation were carried out in MATLAB (R2022a), and the predictive accuracy of different training algorithms was assessed using the correlation coefficient (R), coefficient of determination (R^2^), mean squared error (MSE), root mean squared error (RMSE), and mean absolute percentage error (MAPE). Higher R^2^ values together with lower error metrics generally indicate better model performance.

## 3. Results

The architecture of the artificial neural network was designed with six input variables, a single hidden layer of neurons, and one output. The descriptive statistics of the physicochemical properties measured in broiler litter samples indicated distinct distribution patterns (Table 1). The physicochemical properties of the litter material presented in Table 1 are reported as mean values obtained from three independent broiler flocks. Although moisture content is known to exert a direct influence on NH_3_ concentration and removal, it was not included as an input variable in the artificial neural network models. This decision was based on the experimental design, in which moisture measurements were performed at wider time intervals to ensure sufficient interaction between sodium bisulfate and the litter material. Because all ANN input variables were required to be recorded simultaneously to maintain temporal consistency, moisture content was excluded from the model inputs.

The measured NH_3_ concentrations exhibited a broad range, varying from 0.03 to 67.5 mg kg^−1^. Four distinct dosages, ranging from 2.5% to 10%, were applied in the study. Both dosage and pH values followed a relatively uniform distribution across the experimental range, ensuring balanced representation for the model training. Most measurements of EC and TDS were concentrated at higher values, with relatively few observations at the lower end. NH_3_ removal efficiencies ranged from 0% to 99.8%, while temperatures varied between 18.9 °C and 30.6 °C.

Sodium bisulfate application generally resulted in decreased NH_3_ concentrations and increased treatment efficiency with increasing dosage [38]. The 2.5% dosage group exhibited low efficiency with substantial variability, whereas a marked improvement was observed at the 5.0% level. Higher treatment efficiencies were achieved at the 7.5% and 10.0% dosages. Overall, the distributions were largely homogeneous with few outliers. These results indicate that NH_3_ concentration, EC, and TDS were more strongly affected by outliers, whereas dosage, pH, temperature, and NH_3_ treatment exhibited relatively stable distributions.

The number of neurons in the hidden layer plays a critical role in the development of artificial neural network models. Although there is no definitive criterion for determining the optimal number of neurons, smaller hidden layers are generally preferred to prevent overfitting. An excessive number of neurons may cause the network to memorize the training data, thereby reducing its generalization capacity. Therefore, a systematic empirical evaluation of different neuron configurations was conducted to identify the most suitable network architecture.

In this study, ten neuron configurations (2, 4, 6, 8, 10, 12, 14, 16, 18, and 20) were tested across four datasets. Four training algorithms (trainlm, traincgf, trainscg, and trainbr) were employed to predict NH_3_ removal efficiency (Table 2). Model performance was evaluated using R^2^, MSE, RMSE, and MAPE, and the algorithm that achieved the highest R^2^ and the lowest error indices was considered the most appropriate model.

The findings revealed that the Levenberg–Marquardt algorithm provided the most accurate predictions, achieving its highest performance with 12 neurons (Table 3). The Bayesian model also demonstrated strong predictive ability at 10 neurons, likely due to its probabilistic framework that reduces overfitting by incorporating prior distributions. In contrast, the Fletcher–Reeves and Scaled Conjugate Gradient algorithms achieved their best results at 20 and 14 neurons, respectively, but their overall performance remained less consistent compared to Levenberg–Marquardt and Bayesian approaches. When comparing the overall results, the prediction accuracies ranked in descending order as follows: LM > BR > FR > SCG. Similar trends have been reported in previous studies, where the Levenberg–Marquardt algorithm frequently outperformed other training algorithms in terms of both convergence speed and accuracy. These results collectively suggest that second-order training algorithms, particularly Levenberg–Marquardt, offer significant advantages for neural network modeling of agricultural datasets.

**Figure 5:**
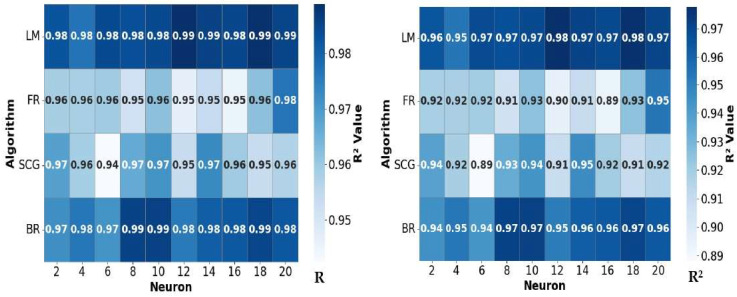

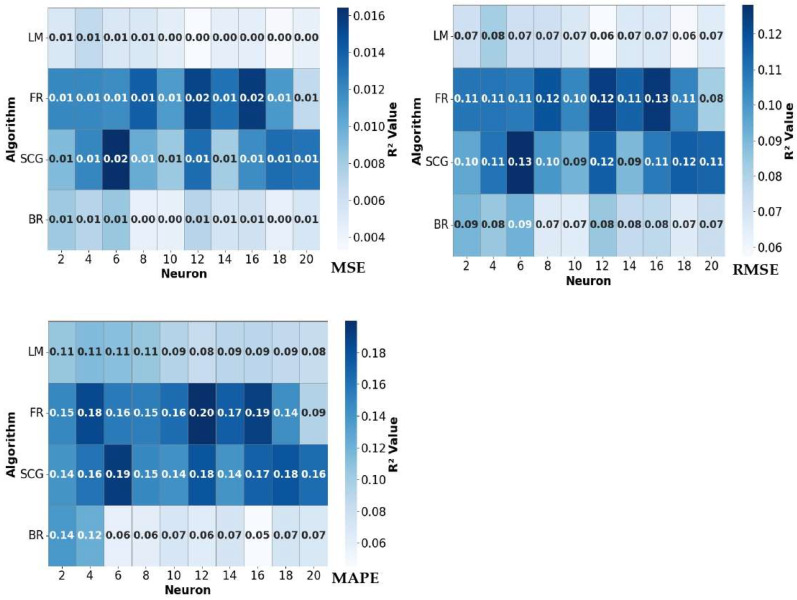
Heatmap visualization of model performance metrics.

The heatmaps in Figure 5 show the model performance of the four algorithms for different neuron numbers. The LM algorithm stands out as the most stable and highest-performing model, exhibiting high R and R^2^ values and low MSE, RMSE, and MAPE across all neuron numbers. The BR algorithm demonstrates similarly high performance but does not reach the minimum error values achieved by LM. In contrast, the FR and SCG algorithms exhibit more variable performance, with decreases in accuracy and increases in error depending on the number of neurons. Specifically, the LM algorithm was identified as having an optimal performance at 12 neurons in terms of maximum accuracy and minimum error. Overall, the Levenberg–Marquardt algorithm with 12 hidden neurons yielded the most accurate predictions (R^2^ = 0.9777, RMSE = 0.0574), followed closely by the Bayesian Regularization algorithm at 10 neurons. These findings suggest that LM is the most suitable algorithm for modeling ammonia mitigation in broiler litter, providing superior prediction accuracy and generalization compared to FR and SCG.

**Figure 6:**
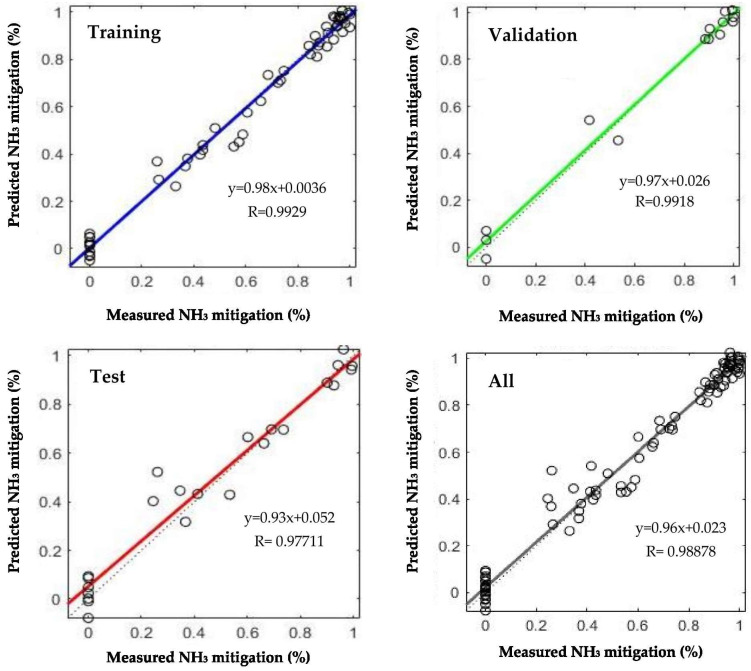
Regression scatter plot of the top-performing Levenberg–Marquardt algorithm.

The Levenberg–Marquardt algorithm demonstrated the highest predictive performance, achieving a maximum regression coefficient of R^2^ = 0.9888 (Figure 6). In Figure 6, the target values on the x-axis represent the measured outputs, whereas the output values on the y-axis correspond to the model-predicted results. By comparison, the R^2^ values obtained for the Fletcher–Reeves, Scaled Conjugate Gradient, and Bayesian algorithms were 0.9765, 0.9727, and 0.9857, respectively. In addition, MAPE values indicated highly accurate predictions (<0.1) for all models except the Scaled Conjugate Gradient algorithm, demonstrating the overall robustness of the developed models [47].

**Figure 7:**
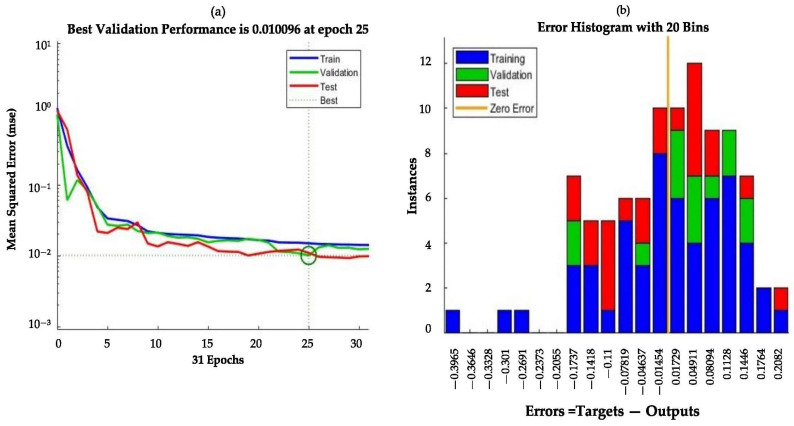
Regression performance (**a**) and error histogram (**b**) of the ANN model (LM, 12 neurons) for NH_3_ removal.

Among the evaluated ANN configurations, the Levenberg–Marquardt algorithm with 12 hidden neurons yielded the highest regression coefficient and the lowest prediction error; therefore, its regression performance and error distribution are presented in Figure 7. The model achieving the highest NH_3_ removal showed a smooth decline in both training and test errors. The best validation performance was observed at epoch 25, where the lowest MSE value was 1.0096 × 10^−2^, as the MSE decreased with increasing numbers of epochs, reaching optimal performance at epoch 25 (Figure 7a). The corresponding error histogram (Figure 7b) revealed that the majority of errors were concentrated between −0.1737 and 0.2082, indicating a dense error distribution. As depicted, most errors were clustered near zero, confirming the model’s high predictive accuracy and robustness. Furthermore, the training, validation, and testing processes followed consistent trends across all predictions, and the close agreement between the validation and test error curves indicates that the model did not suffer from overfitting.

## 4. Discussion

This study evaluated the predictive performance of four different ANN training algorithms for estimating ammonia removal from spent broiler litter treated with sodium bisulfate at various dosages. Among the tested models, the Levenberg–Marquardt algorithm demonstrated the highest predictive accuracy based on regression coefficients and error metrics, using physicochemical inputs including ammonia concentration, additive dosage, pH, electrical conductivity, total dissolved solids, and temperature. This superior performance can be attributed to its second-order optimization nature, which enables faster convergence and higher precision in complex nonlinear datasets [48,49,50].

Previous studies have investigated the effect of sodium bisulfate on ammonia emissions from poultry production [7,38,42] and have applied machine learning methods for ammonia prediction [33,34,51,52]. However, research integrating laboratory-scale evaluations with ANN-based modeling remains limited. While contemporary research in this field predominantly focuses on monitoring and predicting ambient ammonia concentrations within poultry facilities using various machine learning models-such as Random Forest, KNN, and LSTM-in field settings, this study distinguishes itself by predicting the removal efficiency of a specific NH_3_ mitigation treatment (Table 4).

Unlike several studies in the literature that prioritize general model comparisons for concentration tracking, this research provides a granular evaluation of four distinct ANN training algorithms (LM, FR, SCG, and BR) to estimate the performance of a laboratory-scale treatment method. The results demonstrate that the Levenberg–Marquardt (LM) algorithm achieves superior predictive accuracy (R^2^ = 0.9777), surpassing the performance metrics reported in other ANN applications with lower correlation coefficients, such as R^2^ = 0.82. Consequently, by providing a robust data-driven approach for estimating mitigation performance, this study offers a specialized decision-support framework that enhances the predictability of air quality management interventions in poultry production systems.

Our findings demonstrate that artificial neural networks can accurately and rapidly predict ammonia concentrations, offering a reliable and practical tool for poultry management. This approach offers an alternative framework to conventional prediction methods and has the potential to contribute to the improvement of environmental sustainability in poultry production systems. Future studies may expand on this work by incorporating additional ecological and management variables to optimize ammonia mitigation strategies further.

## 5. Conclusions

This study demonstrated that ANN-based prediction provides high accuracy in estimating NH_3_ mitigation efficiency when sodium bisulfate is applied to used broiler litter. Among the four tested algorithms, the Levenberg–Marquardt model showed the best performance (R^2^ = 0.9777, MSE = 0.0033, RMSE = 0.0574, MAPE = 0.0833). These findings confirm that ANNs can reliably and rapidly predict NH_3_ levels in poultry houses, offering a practical tool for improving environmental control and decision-making.

Moreover, the proposed approach can reduce operational costs and time while enhancing the effectiveness of remediation strategies in field applications. Overall, this study highlights the potential of AI-driven predictive modeling as a novel, efficient, and sustainable method for environmental management in modern agricultural systems.

## Figures and Tables

**Table 1:** Statistical summary of litter physicochemical properties across three broiler flocks.

	Inputs						Outputs
	NH_3_ Concentration (mg kg^−1^)	Dosage (%)	pH	EC (μS cm^−1^)	TDS (mg L^−1^)	Temperature (°C)	NH_3_ Treatment (%)
Min	0.03	0.00	1.98	1526.00	766.00	18.85	0.00
Max	67.46	10.00	8.56	3999.00	2000.00	30.55	99.80
Mean	9.54	5.00	5.84	3669.54	1833.47	26.64	58.63
SD	12.33	3.53	1.85	600.65	302.80	3.49	38.36

Min: Minimum, Max: Maximum, Mean: Mean, SD: Standard deviation.

**Table 2:** ANN training algorithms and abbreviations used in this study.

Abbreviation	Algorithm	MATLAB Function
LM	Levenberg–Marquardt	trainlm
FR	Fletcher–Reeves	traincgf
SCG	Scaled Conjugate Gradient	trainscg
BR	Bayesian Regularization	trainbr

**Table 3:** Performance comparison of ANN training algorithms in predicting NH_3_ mitigation efficiency across varying neuron numbers.

Algorithm	Neuron	R	R^2^	MSE	RMSE	MAPE
Levenberg–Marquardt Algorithm (LM)	2	0.9823	0.9649	0.0052	0.0721	0.1058
	4	0.9767	0.9539	0.0068	0.0825	0.1143
	6	0.9841	0.9684	0.0053	0.0728	0.1103
	8	0.9834	0.9671	0.0052	0.0721	0.1058
	10	0.9844	0.9690	0.0046	0.0678	0.0921
	12	0.9888	0.9777	0.0033	0.0574	0.0833
	14	0.9856	0.9713	0.0045	0.0671	0.0899
	16	0.9847	0.9696	0.0045	0.0671	0.0895
	18	0.9883	0.9768	0.0034	0.0583	0.0861
	20	0.9853	0.9709	0.0043	0.0656	0.0829
Fletcher–Reeves Algorithm (FR)	2	0.9585	0.9188	0.0119	0.1091	0.1480
	4	0.9583	0.9183	0.0120	0.1095	0.1849
	6	0.9593	0.9203	0.0117	0.1082	0.1552
	8	0.9519	0.9061	0.0140	0.1183	0.1535
	10	0.9619	0.9253	0.0110	0.1049	0.1638
	12	0.9467	0.8961	0.0154	0.1241	0.1993
	14	0.9541	0.9103	0.0131	0.1145	0.1724
	16	0.9451	0.8931	0.0158	0.1257	0.1900
	18	0.9618	0.9250	0.0111	0.1054	0.1439
	20	0.9765	0.9536	0.0068	0.0825	0.0939
Scaled Conjugate Gradient (SCG)	2	0.9686	0.9381	0.0091	0.0954	0.1400
	4	0.9587	0.9191	0.0121	0.1100	0.1598
	6	0.9424	0.8881	0.0164	0.1281	0.1918
	8	0.9665	0.9342	0.0100	0.1000	0.1474
	10	0.9712	0.9432	0.0083	0.0911	0.1417
	12	0.9535	0.9092	0.0133	0.1153	0.1772
	14	0.9727	0.9461	0.0080	0.0894	0.1403
	16	0.9594	0.9205	0.0117	0.1082	0.1701
	18	0.9538	0.9098	0.0133	0.1153	0.1773
	20	0.9570	0.9158	0.0130	0.1140	0.1635
Bayesian Regularization Algorithm (BR)	2	0.9718	0.9444	0.0082	0.0906	0.1375
	4	0.9762	0.9529	0.0072	0.0849	0.1223
	6	0.9711	0.9430	0.0084	0.0917	0.0581
	8	0.9855	0.9712	0.0044	0.0663	0.0614
	10	0.9857	0.9715	0.0043	0.0656	0.0707
	12	0.9756	0.9518	0.0072	0.0849	0.0631
	14	0.9806	0.9616	0.0057	0.0755	0.0723
	16	0.9816	0.9635	0.0059	0.0768	0.0453
	18	0.9851	0.9705	0.0044	0.0663	0.0734
	20	0.9818	0.9638	0.0054	0.0735	0.0685

**Table 4:** Related work comparison of machine learning studies in poultry production systems.

Study	Environment	Poultry Type	Inputs	Best-Performing Model	Metrics	Key Performance (Best Model)	Main Objective
[22]	Field	Not Specified	Weather data, daily feed consumption, water consumption, number of birds	ANN	R, MSE	R: 0.953, MSE: 3.07 × 10^−3^	Predicting energy consumption of poultry facilities
[31]	Field	Laying hen	Housing system, age of birds, rectal temperature, pulse rate, respiratory rate	ANN	R, R^2^, RMSE	R: 0.983, R^2^: 0.966, RMSE: 0.04806	Predicting heat stress in laying hens
[32]	Field	Broiler	Feeds, electricity, fuel, water, broiler farms, chicks, human labor, machinery parameters	FFBP-ANN *	R^2^, RMSE, MAE	R^2^: 0.936, RMSE: 0.232, MAE: 0.019	Estimating total energy equivalent of broiler meat
[33]	Field	Broiler	Air temperature, relative humidity, air velocity	ANFIS-SC *	R^2^, RMSE, MRPE	R^2^: 0.858, RMSE: 1.130 ppm, MRPE: 4.032%	Predicting NH_3_ concentration in poultry farms
[51]	Field	Broiler	Air temperature, relative humidity, air velocity	RF-WT *	MAE, R	MAE: 0.548 ppm R: 0.976	Developing a hybrid model for NH_3_ prediction
[52]	Field (smart farm)	Laying hen	CO_2_, CH_4_ and NH_3_ concentrations	LSTM *	R^2^, MAE, RMSE	MSE: 35.2 R^2^: 0.87	Measuring gas concentrations using AI and IoT-based systems
[53]	Field	Broiler	Housing condition, age week, temperature, ventilation, NH_3_ concentration	Multilayer Perceptron	Accuracy: 98%	Multilayer Perceptron	Assessing ammonia exposure risk and its relationship with health outcomes in broiler housing
[54]	Field	Laying hen	Temperature, relative humidity, ventilation, air velocity, number of animals, ammonia concentration	ANN	R, R^2^, RMSE, MAE, MAPE	R^2^: 0.9741, MSE: 0.0027; RMSE: 0.0521; MAE: 0.0236; MAPE: 0.0536	NH_3_ emission modeling in laying hen farm
This study	Lab	Broiler	NH_3_ concentration, dosage, pH, EC, TDS, temperature	ANN	R, R^2^, RMSE, MAE	R: 0.9888, R^2^:0.9777 MSE: 0.0033, RMSE: 0.057; MAPE: 0.0833	Predicting NH_3_ mitigation efficiency under controlled conditions

* FFBP-ANN: feed forward back propagation artificial neural network model; ANFIS-SC: Integrated adaptive neuro-fuzzy inference systems with subtractive clustering; RF-WT: random forest-wavelet transform; LSTM: Long Short-Term Memory.

## Data Availability

The raw data supporting the conclusions of this article will be made available by the authors on request.
